# Therapeutic Vaccines for Head and Neck Squamous Cell Carcinoma and Nasopharyngeal Carcinoma

**DOI:** 10.3390/vaccines14040321

**Published:** 2026-04-03

**Authors:** Michael Baliton, Duha Alfatlawi, Shirin Attarian, Rupali Nabar, Victoria Villaflor

**Affiliations:** 1 UC Irvine Medical Center, Orange, CA 92868, USA; mbaliton@hs.uci.edu (M.B.);; 2 UC Irvine School of Medicine, Irvine, CA 92617, USA; dalfatla@hs.uci.edu; 3 Division of Hematology/Oncology, Department of Medicine, UCI Health Chao Family Comprehensive Cancer Center, Orange, CA 92868, USA

**Keywords:** head and neck squamous cell carcinoma (HNSCC), Epstein–Barr virus (EBV), human papilloma virus (HPV), immunotherapy, therapeutic vaccine, mRNA vaccine, peptide vaccine

## Abstract

**Background and Objectives:** Recent evidence demonstrates additional survival benefit of immune checkpoint inhibitor (ICI) therapy in the treatment of head and neck squamous cell carcinoma (HNSCSC) and nasopharyngeal carcinoma (NPC). However, overall outcomes remain relatively stagnant despite this significant progress. Therapeutic vaccines are a promising adjunct to existing systemic therapy strategies in HNSCC and NPC. This review aims to summarize current evidence, review ongoing studies, and discuss areas of opportunity and potential future directions of vaccine therapy in this space. **Methodology:** A comprehensive review of the current literature was conducted through database searches on PubMed and ClinicalTrials.gov. Studies were stratified by tumor type, vaccine delivery platform, and early versus recurrent metastatic (RM) disease. **Results:** Therapeutic vaccines in combination with ICI for HPV-associated HNSCC have shown the most promise, though modest. Vaccine delivery in HPV-negative HNSCC and NPC are still in early development. Integration of therapeutic vaccines across these tumor types is challenged by immune escape mechanisms, lack of viable targets, and tumor heterogeneity. **Conclusions**: Early data suggest that therapeutic vaccines in combination with ICIs may offer additional benefit in the treatment of HNSCC and NPC, especially in RM HPV-associated HNSCC. Future efforts should validate these early findings through phase 3 trials. Data regarding therapeutic vaccines combined with chemotherapy or radiation is limited but may also provide additional benefit.

## 1. Introduction

Head and neck cancers comprise malignancies arising from the mucosa of the oral cavity, oropharynx, larynx, and hypopharynx. Specifically, head and neck squamous cell carcinoma (HNSCC) accounts for over 90% of head and neck cancers and is the seventh-most common malignancy worldwide with 890,000 new cases and 450,000 deaths annually. HPV-associated HNSCC is driven predominantly by high-risk HPV-16 and most commonly arises in the oropharynx [1,2]. In contrast, HPV-negative tumors are linked to chronic exposure to tobacco and alcohol and are associated with more aggressive disease and poorer outcomes. Nasopharyngeal carcinoma (NPC) distinctly arises from the nasopharyngeal mucosa and has a high incidence in Asia. According to GLOBOCAN 2022 data, China alone accounted for 51,010 incident NPC cases in 2022, contributing to 42.3% of the 120,434 total incident cases of NPC globally [3]. Major risk factors for NPC include Epstein–Barr virus (EBV) infection and environmental exposures to tobacco smoke, formaldehyde, and consumption of salted and preserved foods [4].

Recent milestones in immune checkpoint inhibitor (ICI) therapy for head and neck cancer have been promising and support the early integration of immunotherapy in HNSCC and NPC.

The landmark KEYNOTE-048 trial established PD-1 blockade as first-line (1L) therapy in unresectable recurrent metastatic (RM) HNSCC, indicating superior overall survival (OS) compared to cetuximab with chemotherapy among patients with positive PD-L1 expression [5]. In patients with resectable disease, the NIVOPOSTOP study determined that postoperative cisplatin and radiation with concomitant and maintenance nivolumab improves disease free survival in locally advanced tumors [6]. Published data from the KEYNOTE-689 trial showed that the addition of perioperative pembrolizumab improved event-free survival compared to standard chemoradiotherapy [7].

For patients with RM NPC, the JUPITER-02 trial reported improved median progression-free survival (PFS) and OS in patients treated with toripalimab plus gemcitabine-cisplatin compared to chemotherapy alone [8]. There are several ongoing trials with early data also indicating the benefit of adding ICI in the definitive treatment of locoregionally advanced NPC [9,10,11]. Subsequent trials are investigating the optimal ICI agent, sequencing, combination strategies with chemotherapy and radiation, and duration of therapy as well as refining patient selection and timepoints. [12,13,14,15,16].

However, despite these advances, survival rates for advanced HNSCC and NPC remain relatively stagnant. There is a need for novel therapeutic approaches that provide effective responses and durable disease control.

Vaccine therapy in HNSCC and NPC is an emerging treatment strategy that may provide additional benefit in tandem with current treatment paradigms. This review will explore the underlying mechanisms, current data, challenges, and future directions of therapeutic vaccines amid the rapidly evolving treatment landscape of HNSCC and NPC.

## 2. Methodology

### 2.1. Search Strategy

Extensive keyword-based searches were conducted on PubMed and Google Scholar for this targeted literature review. A list of the applied search terms is included in Supplementary Table S1. Searches were performed between November 2025 and January 2026. Reference lists for relevant publications were screened to identify additional resources. The ClinicalTrials.gov database was manually queried for relevant clinical trials and relevant results were reported when available.

### 2.2. Selection Criteria

**Figure 1:**
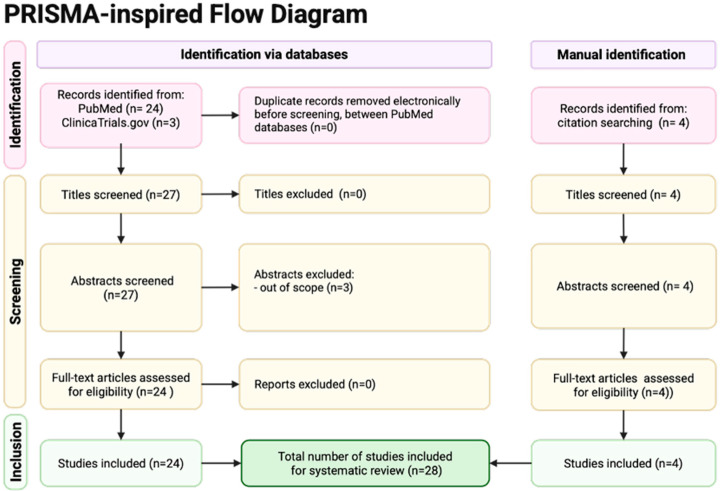
PRISMA-inspired flow diagram of literature identification. The authors searched the PubMed and ClinicalTrials.gov databases for phase 1–3 randomized clinical trials using key search terms. Citations were manually reviewed and four additional studies were included for screening. A total of 29 full text articles were assessed for eligibility. Reports were excluded if the study did not focus on HNSCC or NPC or if subgroup data for these tumor types were not available. A total of 25 sources were included for review.

This review includes phase 1–3 clinical trials that have evaluated therapeutic vaccines for HNSCC and NPC between 2007 and 2025 (Figure 1). Searches were limited to clinical studies published in English and were further stratified by treatment setting. Studies were excluded if the objective of the study did not focus on outcomes in HNSCC and NPC or if subgroup data was not available. Therapies for RM disease were delineated as 1L or second-line (2L) or greater treatment. Therapies in the curative setting, including neoadjuvant/induction, definitive, and adjuvant treatments, were also included. Basic science studies were utilized to contextualize the mechanism of action of each vaccine delivery class and each individual therapy, but were not included in the final review and analysis. The authors discussed the compiled publications, and the senior authors provided insight on the most clinically relevant studies.

## 3. HPV-Positive HNSCC Vaccines

The goal of therapeutic HPV vaccines is to induce an antigen-specific CD4-positive and CD8-positive T-cell response against HPV-driven tumor cells, which have adapted immune evasion mechanisms, by often targeting E6/E7 [17]. Prophylactic HPV vaccines target the L1 capsid protein; however, L1 is generally not expressed in established HPV-driven cancers and are therefore not therapeutic [17,18].

HPV-positive HNSCC tumors are histologically characterized by an inflammatory tumor microenvironment (TME) enriched with tumor-infiltrating lymphocytes (TILs) [19]. Samples of T-cell infiltrate in HPV-positive tumors demonstrate greater intratumoral T-cell receptor diversity compared to that of HPV-negative tumor-infiltrating lymphocytes, which is a marker of greater immunologic activity [20]. The HPV-positive TME also exhibits relatively higher PD-L1 expression that can be targeted by PD-1/PD-L1 blockade [21]. This molecular profile predicts favorable responses to immunomodulatory therapy [22,23]. In this section, we outline recent vaccines focused on enhancing antitumor activity in combination with ICIs, targeted therapy, or chemotherapy.

Therapeutic HNSCC vaccines have been evaluated through various platforms. We have provided a table summary of several key clinical trials and current data to date in Appendix A. We aim to highlight top-line results in HPV-positive HNSCC vaccine therapy.

### 3.1. Peptide-Based Vaccines

**Figure 2:**
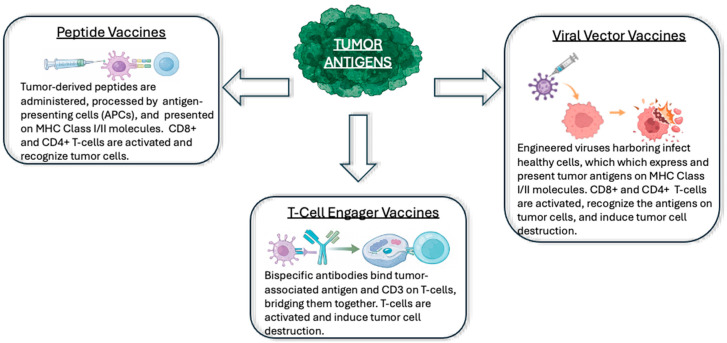
Mechanisms of Therapeutic Cancer Vaccines. Therapeutic vaccines utilize tumor antigens to induce an immune response against cancer cells and can be delivered through different platforms including peptide or mRNA, viral vectors, and T-cell engager molecules.

Peptide vaccines deliver synthetic, tumor-specific epitopes that are cellularly processed and cross-presented to immune cells leading to a targeted immune response (Figure 2). As vaccine integrity may be compromised by cellular proteasome processing, the length and structure of these vaccines are carefully designed to ensure proper antigen presentation [24,25]. Peptide-based vaccines often require adjuvants to improve antigenicity or specialized delivery systems for improved uptake [24].

HPV proteins E6 and E7 have been central targets for therapeutic vaccines. Both proteins are essential for viral replication and drive malignant transformation by inhibiting tumor suppressor proteins p53 and Rb, respectively [26,27,28]. These tumor-specific antigens are absent in normal cells but are expressed on virally infected tumor cells, which makes them highly immunogenic [29,30]. E6 and E7 have been shown to elicit robust antigen-specific CD4-positive and CD8-positive T-cell responses and durable humoral immunity, and have been marketed as ideal therapeutic candidates for vaccine development [31,32].

#### 3.1.1. Peptide Vaccines in RM HPV-Positive HNSCC

Peptide vaccines for HPV-positive disease have been most studied in the RM setting. For example, ISA101 is a long peptide vaccine targeting E6 and E7 that demonstrated clinical activity in combination with nivolumab in patients with incurable HPV-associated cancers (NCT02426892) [33,34]. OpcemISA (NCT03669718) was a phase 2 trial of ISA101 plus cemiplimab in first-line or second-line treatment in RM disease [35]. While this combination did not improve ORR for all participants, those with PD-L1 CPS ≥ 1 demonstrated improved ORR from the addition of ISA101 to ICI. Higher CPS scores were positively correlated with greater ORR [35]. This benefit was most pronounced in patients with PD-L1 CPS ≥ 20 and received all three vaccine doses. The efficacy of this same combination will be evaluated in a phase 2 study (NCT04398524) for patients with RM OPC but had progression of disease after treatment with anti-PD-1 blockade [36].

PDS0101 is an HLA-unrestricted, peptide vaccine containing E6 and E7 that is delivered with an immune-stimulating cationic lipid, R-DOTAP. VERSATILE-002 (NCT04260126) was a single-arm phase 2 trial assessing PDS0101 in combination with pembrolizumab as first-line therapy in the RM setting [37]. This combination showed significant clinical activity across all participants and those with PD-L1 CPS ≥ 20 had higher response rates [37]. The efficacy of PDS0101 plus pembrolizumab compared to pembrolizumab monotherapy is now being evaluated as first-line therapy in patients with RM disease and with PD-L1 ≥ 1 in an ongoing phase 3 trial, VERSATILE-003 [38].

A combination of PDS0101, bintrafusp alfa, and NHS-IL12 will be studied in a phase 1/2 clinical trial (NCT04287868). Bintrafusp alfa is a dual-action fusion protein that blocks PD-L1 and sequesters TGF-β from the tumor microenvironment, whereas NHS-IL12 is a therapy that combines necrosis-directed human IgG linked to recombinant IL-12. The overall goal of this approach is to downregulate tumor-mediated, immunosuppressive signaling while inducing a Th-1 response to overall enhance tumor cytotoxicity [39,40,41].

#### 3.1.2. Peptide Vaccines in Early-Stage HPV-Positive HNSCC

Given these promising results, peptide vaccines are also being evaluated in the early disease setting. In newly diagnosed patients with locally advanced disease, ISA101b is also being evaluated in intermediate-risk oropharyngeal tumors in a phase 2 study combining ISA101b, pembrolizumab, and concurrent cisplatin with radiation as definitive treatment (NCT04369937) [42]. This also includes a phase 2 trial of PDS0101 plus pembrolizumab versus pembrolizumab monotherapy as neoadjuvant treatment in locally advanced disease (NCT05232851) [43]. Interim analysis after two cycles of neoadjuvant treatment demonstrated that 50% of participants receiving combination therapy met the primary endpoint of 50% reduction in ctDNA compared to 0% of participants in the ICI monotherapy arm [43].

### 3.2. Viral Vector Vaccines

Viral vaccines utilize non-oncolytic, replication-deficient virus vectors to deliver genetic material that encode for target antigens. Once expressed, the antigen is processed for cell-surface presentation and elicits a targeted immune response [44,45].

#### 3.2.1. Viral Vector Vaccines in RM HPV-Positive HNSCC

HB-201 and HB-202 are genetically engineered live-attenuated arenavirus vectors derived from lymphocytic choriomeningitis virus (LCMV) and Pichinde virus (PICV), respectively [46]. Vectors preferentially infect antigen-presenting cells and deliver RNA encoding a non-oncogenic HPV16 E7/E6 fusion antigen, thereby priming and expanding HPV-specific T-cell immunity. HB-200 comprises an alternating sequence of HB-201 and HB-202. [46,47].

A phase 2 study of HB-200 plus pembrolizumab as 1L treatment in the RM setting showed a meaningful increase in antigen-specific CD8-positive T-cell responses among 71% of participants (NCT04180215) [48]. Disease response rates were promising, especially among participants with PD-L1 CPS ≥ 20, which included 17 patients who achieved a complete response. Participants who demonstrated no evidence of disease following definitive treatment but had detectable HPV ctDNA levels after 3 months will be enrolled into a phase 2 study to assess whether HPV ctDNA measurements can predict disease recurrence (NCT06373380) [49]. AVALON-1 will be a phase 3 trial of HB-200 plus checkpoint inhibitor therapy as 1L treatment in the RM setting [48].

Other viral vaccines have been studied in HPV-driven tumors however with limited success. A phase 2 trial evaluated HPV-associated tumors treated with PRGN-2009, a gorilla Adenovirus-based vaccine that contains recombinant DNA encoding for HPV E6 and E7 epitopes, in combination with bintrafusp alfa, (NCT04432597) [50,51,52]. There was only modest clinical benefit in a subgroup analysis of patients with RM HNSCC treated with bintrafusp alfa, which had an ORR of 13% (NCT02517398) [53].

#### 3.2.2. Viral Vector Vaccines in Early-Stage HPV-Positive HNSCC

Viral-based vaccines in early-stage disease are limited but are gaining traction. For example, TARGET-HPV is an ongoing phase 1/2 clinical trial of neoadjuvant HB-200 in combination with carboplatin and paclitaxel for locally advanced disease (NCT05108870). Preliminary results are promising and show a deep response rate of 81% and 93% in patients treated with higher doses [54].

### 3.3. Nucleic Acid-Based Vaccines in HPV-Positive HNSCC

Like peptide vaccines, nucleic acid-based vaccines contain recombinant genetic material that are cellularly processed and recognized by the immune system.

The mRNA vaccine BNT113 encodes for HPV E6/E7. The phase II/III AHEAD-MERIT trial studied BNT113 in combination with pembrolizumab versus pembrolizumab monotherapy as 1L treatment in patients with RM disease with positive PD-L1 expression (NCT04534205) [55]. Preliminary data showed positive survival benefit with combination therapy and has been granted fast track designation by the FDA [56].

INO-3112 is a DNA plasmid encoding for viral E6, E7, and recombinant IL-12, which was previously shown to have adequate T-cell activity in patients with HPV-associated tumors [57]; however, a subsequent phase 1/2 study of INO-3112 plus durvalumab in RM HNSCC was terminated due to limited efficacy in this subgroup (NCT03162224) [58].

### 3.4. Prime-Boost Vaccines in HPV-Positive HNSCC

Heterologous prime-boost vaccines utilize a two-step approach. The immune system is primed with an initial exposure to a tumor antigen and subsequent exposures enhance the immune response, oftentimes in the form of another vaccine platform [59]. A single-arm, phase 2 trial of pembrolizumab, pBI-11, and TA-HPV as 1L therapy for RM HPV-positive HNSCC is currently recruiting participants (NCT05799144) [60]. The goal of this treatment is to introduce a DNA-based plasmid, pBI-11, that encodes for an E6/E7 fusion protein linked to heat shock protein 70 (HSP70), which is a potent dendritic cell activator [61]. This followed by TA-HPV therapy which is a viral vector utilizing modified vaccinia containing recombinant E6/E7 DNA [62]. This heterologous vaccine strategy in combination with ICI is thought to provoke a more durable immune response compared to a homologous therapeutic vaccination.

### 3.5. Bacterial Vector Vaccines in HPV-Positive HNSCC

Bacteria can be genetically modified to intracellularly infect APCs and induce expression and cross-presentation of target antigens; however, success has been limited in the treatment of HNSCC. A phase 2 study evaluated pre-operative treatment with ADXS11-001, a bioengineered, live-attenuated strain of Listeria monocytogenes that secretes a fusion protein HPV E7-LLO, compared to surgery alone (NCT02002182) [63]. While the treatment increased the HPV-specific T-cell response in 33% of treated patients, 55% developed grade 3 or 4 adverse events, including hypertension, dysphagia, vomiting, and failure to thrive [64]. Follow-up phase 1 trials evaluating ADXS11-011 with or without durvalumab (NCT 02291055, NCT01598792) respectively, were complicated by significant toxicity [65,66]. 

### 3.6. T-Cell Engager Vaccines in HPV-Positive HNSCC

T-cell engager vaccines are bispecific antibodies that simultaneously bind T-cell activation markers such as CD3 with a target antigen on tumor cells. This physically bridges T-cells to their targets, triggers a potent, and specific cytotoxic response, and bypasses the need for conventional antigen presentation [67,68].

#### 3.6.1. T-Cell Engager Vaccines in RM HPV-Positive HNSCC

CUE-101 is a T-cell engager fusion protein that consists of an HLA-A02:01 molecule loaded with E7 epitopes linked to affinity-attenuated IL-2 molecules [69]. This novel design promotes induction and expansion of E7 antigen-specific CD8-positive T-cells to limit off-target T-cell activation and reduce systemic IL-2 toxicity. CUE-101-01 (NCT03978689) is a current study evaluating CUE-101 as monotherapy or in combination with pembrolizumab as first-line or subsequent treatment in RM HPV-positive HNSCC [69]. Participants receiving CUE-101 plus pembrolizumab as 1L treatment showed promising response rates from preliminary data and will need further validation in larger phase III studies.

Response rates were significantly lower among those who were pre-treated with ICI or platinum-based chemotherapy and assigned to receive CUE-101 monotherapy. Given that cancer-directed therapy selects for treatment-resistant tumor cells, tumors previously exposed to ICI or chemotherapy may respond poorly to rechallenge if the subsequent therapy is the same. While these results are preliminary, CUE-101-01 may suggest that the addition of CUE-101 to ICI may not change this precedent in pre-treated patients but may be an effective strategy as 1L therapy.

#### 3.6.2. T-Cell Engager Vaccines in Early-Stage HPV-Positive HNSCC

Future data analysis may also clarify the efficacy of this approach when stratified by PD-L1 expression levels and when integrated in earlier lines of treatment. A current phase 2 study will evaluate the efficacy and timing of neoadjuvant CUE-101 through three different dosing schedules prior to definitive therapy (NCT04852328) [70].

## 4. HPV-Negative HNSCC Vaccines

In comparison to HPV-positive disease, HPV-negative HNSCC lack immunogenic viral-associated antigens and have high rates of recurrence [71,72]. Approximately 40–60% of HPV-negative tumors will recur, with locoregional recurrence more common than distant metastasis [73]. Vaccine development in this area has focused and primarily targeted tumor-associated antigens, which are proteins that regulate cell growth and division but are overexpressed or genetically altered in cancer cells such as p53, MUC-1, and survivin. This reflects early data from a smaller studies, but further efforts in personalized neoantigen therapy may offer potential benefit.

### 4.1. Therapeutic Vaccines in RM HPV-Negative HNSCC

Personalized neoantigen vaccines in HPV-negative tumors are in clinical early development. Each tumor is molecularly sequenced, and unique tumor neoantigens are selected, manufactured, and reintroduced to induce a precise immune response that is specific to the tumor [74].

TG4050 is a modified Vaccinia Ankara viral-based vaccine which has shown clinical activity as maintenance therapy for patients with locally advanced HNSCC who have achieved complete remission following surgery and adjuvant therapy in a randomized, open label, phase I trial. The vaccine was well tolerated. At a median follow up of 28.5 months, the recurrence rate in the treatment arm (*n* = 16) was 0% compared to 19% in the control arm (NCT04183166) [71,72]. Notably, the participants in the treatment arm remained disease-free despite the challenging histologic landscape that HPV-negative tumors exhibit. While this data is preliminary, these early findings may be a positive signal for personalized neoantigen vaccines and will require further investigation in larger phase III studies. Future efforts may trial in the RM setting, in earlier lines of therapy, or in combination with chemotherapy or immunotherapy.

Melanoma-associated antigen A3 (MAGE-A3) is a tumor-specific antigen that is aberrantly expressed in several tumor types including HNSCC, and high expression is associated with poor prognosis [75]. This cancer-testis antigen protein binds to E3 ubiquitin-ligases and downregulates p53-mediated apoptosis, which confers resistance to conventional systemic therapy. MAGE-A3 also induces the epithelial–mesenchymal transition within tumor cells ultimately leading to metastasis and invasion of distant tissues [76,77,78].

A phase I trial (NCT00257738) assessed the efficacy of MAGE-A3 vaccines in both HPV 16 positive and MAGE-A3 positive tumors in the RM setting [79]. While the therapy induced antigen-specific CD8-positive T-cell responses in most participants (80% in the HPV 16 group and 67% in the MAGE-A3 positive group), all but one participant had progressive disease at their first imaging evaluation.

New York esophageal squamous cell carcinoma 1 (NYO-ESO-1), a cancer-testis antigen that is expressed in several tumors including HNSCC [80]. Like MAGE-A3, NY-ESO-1 prevents ubiquitination and inhibits cell detachment-induced apoptosis, thereby promoting dysregulated cell survival and tumor metastasis [81,82]. Its expression is associated with more immunosuppressive tumor microenvironments and overall poor prognosis [83]. Previous studies of NY-ESO-1 vaccines in other tumor types have shown its capacity to induce antigen-specific CD8-positive T-cell responses [84].

However, specific data regarding its efficacy in HNSCC is limited to a case report of a patient with RM HNSCC treated with nivolumab who had measurable NY-ESO-1-specific CD8-positive T cell response during disease stability and diminished as the disease progressed [85].

HNSCC tumors have also been shown to overexpress EGFR in 90% of cases. CIMAvax is a recombinant human EGF conjugated to a carrier protein that induces neutralizing anti-EGF antibodies, thereby depleting circulating EGF and blocking EGF–EGFR interaction to dampen EGFR-driven proliferative signaling. This therapy is currently being evaluated in a phase I/II trial in combination with nivolumab or pembrolizumab for NSCLC and RM HNSCC [86].

### 4.2. Therapeutic Vaccines in Early-Stage HPV-Negative HNSCC

A phase II, open label, nonrandomized trial of a vaccine containing LY6K, CDCA1, and IMP3 peptides was administered to participants with locally advanced HNSCC but also enrolled patients with RM HPV-negative disease who met criteria [87]. This multi-peptide approach attempts to overcome tumor cell escape and heterogeneity. The trial showed a modest but statistically significant OS benefit in the A24-positive subgroup compared to the A24-negative subgroup (median survival time 4.9 vs. 3.5 months) [87].

Survivin-2B is an inhibitor of apoptosis that is highly expressed in HNSCC, but absent in normal tissue [88]. Higher levels of expression correlate with resistance to chemotherapy, radiation, and overall poor prognosis [89]. A phase I study assessed the efficacy of a peptide vaccine against this protein in 11 patients, but only showed marginal clinical response [90].

Autologous immune cell vaccine therapy utilizes APCs derived from each participant by engineering them in vitro with molecules that induce tumor cell apoptosis. Intranodal administration of mutant p53, a tumor suppressor, loaded onto autologous dendritic cells showed a favorable 3 year OS compared to chemoradiation alone (88% vs. 70%) in a phase I study, but this was limited by under-enrollment (NCT00404339) [91].

### 4.3. Limited Efficacy of HPV-Negative HNSCC Vaccines

Compared to HPV-positive HNSCC tumors, there is a paucity of data showing the efficacy of therapeutic vaccines in the HPV-negative setting, which calls for improved discovery of targetable tumor antigens. Treatment in this setting is challenging due to the immunohistologic profile of these tumors. The relatively lower levels of cytotoxic tumor lymphocyte infiltration pose a fundamental challenge of developing vaccines for HPV-negative disease which would theoretically lead to limited response to immunotherapy.

This underscores the importance of finding other therapeutic targets with viable clinical activity. Ideally these candidates demonstrate utility across several domains such as predicting overall prognosis, tracking treatment response, and monitoring disease recurrence. Personalized neoantigen vaccines such as TG4050 may overcome these barriers by nature of their novel design; however, this approach is still in early development.

Similar to HPV-positive HNSCC treatment strategies, therapeutic vaccines in this space should focus on combining immune checkpoint blockade or other standard treatments to enhance immunogenicity and antitumor activity. Overall, the treatment of HPV-negative HNSCC presents an opportunity for further development and highlights the need for the discovery and validation of biomarkers and novel strategies that augment current treatment paradigms.

## 5. Overview of EBV-Positive NPC Vaccines

### 5.1. Pathophysiology of EBV-Positive NPC

**Figure 3:**
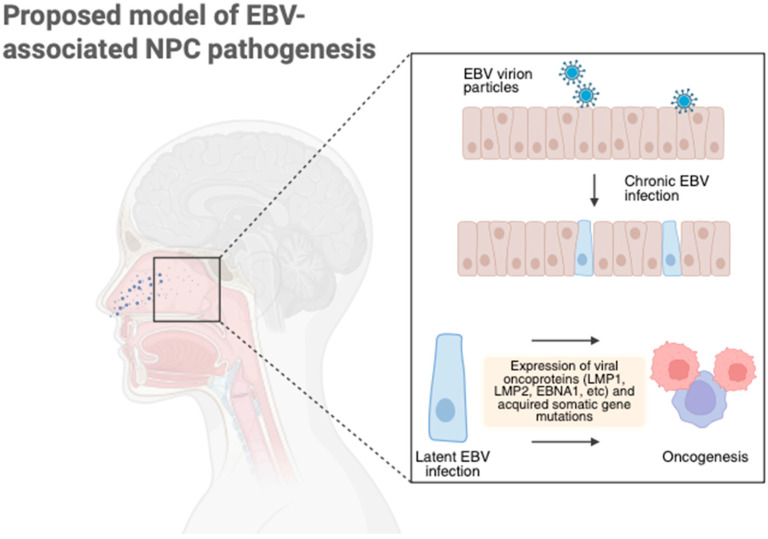
Epstein–Barr virus (EBV) infects normal nasopharyngeal epithelium which leads to chronic EBV infection. The virus expresses viral oncoproteins in latency, including LMP1, LMP2, and EBNA1. Acquired gene somatic mutations in TP53, RAS, and several cell cycle regulatory pathways results in oncogenesis and malignant transformation.

Epstein–Barr virus (EBV) associated nasopharyngeal carcinoma (NPC) arises from malignant transformation of nasopharyngeal epithelial cells due to persistent EBV infection. This mainly occurs through a latency II pattern that is characterized by the expression of EBNA1, latent membrane protein 1 (LMP1), latent membrane protein 2 (LMP2), and multiple non-coding RNAs [80] (Figure 3).

Each of these viral proteins serves an essential role in the development of NPC. EBNA1 maintains and replicates viral episomes while also modulating host chromosomal stability and altering DNA repair responses. LMP1 functions as a constitutively active CD40 mimic and activates the NF-κB, JAK/STAT, MAPK, and PI3K/AKT pathways. These pathways drive proliferation, apoptotic resistance, epithelial–mesenchymal transition (EMT), angiogenesis, and immune escape [92,93]. LMP2 enhances epithelial survival, promotes dedifferentiation, and disrupts normal cell adhesion and polarity [94]. EBV also encodes microRNAs, reprograms host gene expression, suppresses antigen processing and presentation pathways, and shapes an immunosuppressive tumor microenvironment. Together, these drivers and changes in the microenvironment produce a genomically stable and immune-evasive cancer that is biologically dependent on the EBV latency proteins. As a result, EBV latency proteins are a compelling target for immunotherapy and therapeutic vaccine development in EBV-positive NPC.

### 5.2. Ideal Immunotherapy Targets in EBV-Positive NPC

In contrast to the many tumor neoantigens that vary across patients, EBV antigens in NPC are relatively genetically conserved and expressed across most undifferentiated non-keratinizing NPC tumors [95,96]. This stability provides a shared antigenic supply of targets that is suitable for therapeutic vaccine development [97]. These antigenic proteins contain many epitopes that are recognized by CD4-positive and CD8-positive T-cells. Moreover, EBV antigens seem to be tumor-specific, constitutively expressed in NPC cells but absent in the surrounding nasopharyngeal epithelium [92]. These advantages make EBV-positive NPC an ideal space for antigen-targeted immunotherapy, providing a clear rationale for vaccine development.

Additionally, the clinical impact of targeting EBV-associated antigens is demonstrated in the phase III JUPITER-02 trial which highlighted the success of immune checkpoint blockade in EBV-positive NPC. The trial revealed that PD-1 inhibition significantly improved outcomes when coupled with first-line chemotherapy [98]. It is clear that EBV-positive NPC is immunologically responsive and that developments in vaccines that increase in T-cells targeting EBV antigens may work well with checkpoint inhibition to further enhance antitumor therapy.

### 5.3. Current Vaccine Development in EBV-Positive NPC

Despite the potential, vaccine development for EBV-positive NPC has not progressed as quickly as other immunotherapeutic approaches. There have only been a few early trials with limited data available. Some early clinical vaccine programs have explored therapeutic avenues with EBNA1 and LMP2 as targets. Using peptide or dendritic cell platforms, these programs were designed as small human trials with the goal of evaluating safety and demonstrating antigen-specific immunogenicity. Results confirmed that EBV antigens could be viable targets; however, limitations included short-lived vaccine-induced immunity and modest clinical efficacy. Additionally, technology at that time lacked optimized adjuvants, advanced antigen-presentation systems and the modern delivery vectors capable of generating strong CD8-positive cytotoxic responses [99].

Another EBV-positive NPC vaccine trial attempted to employ the use of an MVA vector. MVA-EL encodes a fusion protein of EBNA1/LMP2 and has been tested in multiple phase I studies in Hong Kong and the UK. These trials demonstrated that MVA-EL is safe, well tolerated, and can significantly expand the EBNA1-specific and LMP2-specific CD4^+^ and CD8^+^ T-cell responses in patients with previously treated NPC [100,101]. However, this study came with a few notable limitations. It did not assess evidence of durable tumor control or reduced relapse. Additionally, the authors of the study noted that the vaccine may have boosted preexisting memory T-cells rather than generation of novel T-cell responses since many of the patients in the study exhibited low-level antigen-specific responses before being vaccinated. Finally, the assays provided a general readout, did not discriminate between CD4-positive and CD8-positive responses and did not guarantee that those T-cells would traffic to tumor sites or infiltrate residual tumor cells. Nonetheless, there was an observed direct correlation between dose administered and EBNA1/LMP2 response, allowing the highest dose to be recommended for administration in future Phase II trials.

A recombinant adenoviral vaccine, Ad-ΔLMP1-LMP2, encoding for truncated EBV antigens has also been studied as a potential therapeutic avenue for EBV-positive NPC [102]. This design aimed to use the strong immunogenicity of adenoviral vectors while minimizing the oncogenic risk of LMP1′s signaling domains. This phase II study was well tolerated, induced a delayed hypersensitivity response in 9/12 patients, and clinical responses in 3/12 patients. However, definitive clinical benefit was limited, and tumor-infiltrating immune responses were difficult to evaluate.

Overall, data for EBV-positive NPC is still in its early stages. Without appropriately immunogenic tumor-associated antigen targets, the efficacy of therapeutic vaccines in this setting remains unclear. These obstacles present future opportunities to find innovative vaccine targets.

## 6. Discussion: Challenges and Future Directions

### 6.1. Tumor Escape Mechanisms

HNSCC and NPC exhibit complex molecular heterogeneity which largely impacts prognosis and treatment response [103]. Furthermore, the immune landscape varies between tumor types, with a particular distinction between tumors driven by HPV or EBV infection compared to relatively less immunogenic, non-virally driven tumors.

Like other cancers, tumors of the HNSCC and NPC evade the immune system through several mechanisms including upregulation of immune checkpoints, overexpression of immunosuppressive cytokines, T-cell exhaustion, and the loss of T-cell effector functions. Overcoming these challenges highlights the need for novel biomarkers, the discovery of effective therapeutic targets, and the development of effective vaccine delivery systems. Future efforts in this space should consider the synergistic potential of multimodal therapy regimens and guide treatment decisions regarding optimal patient selection.

### 6.2. Rationale for Combination Therapy

There are currently no FDA-approved therapeutic vaccines for the treatment of head and neck cancer to date. In addition to assessing the efficacy of this novel strategy, current research centers around its optimal integration within current treatment paradigms.

Therapeutic vaccines for HNSCC and NPC have largely been studied in combination with chemotherapy or immunotherapy. Vaccine monotherapy has been thought to have limited clinical activity especially in the treatment of widespread RM disease [104]. The limitations of this approach are currently not completely understood; however, this is likely multifactorial due to several mechanisms such as tumor escape, molecular heterogeneity, and the suppressive tumor microenvironment, which highlights the need for multiple modalities of systemic treatment.

The combination of both vaccine therapy with ICIs is grounded in the complementary mechanisms by which each approach targets the tumor immune landscape. While vaccines induce and expand tumor-specific T-cell responses, their efficacy is limited by the immunosuppressive tumor environment. Immune checkpoint therapy relieves some of these inhibitory signals and thereby amplifies vaccine-mediated antitumor activity [104].

There is limited evidence to support the efficacy of therapeutic vaccines combined with standard therapies, such as chemotherapy and radiation, in HNSCC and NPC. However, this may be a reasonable approach and a potential area of future interest as both chemotherapy and radiation enhance tumor antigenicity, upregulate MHC I presentation, and enhance antigen presentation [105].

### 6.3. Limitations in HPV-Negative and EBV-Positive Disease

At this juncture, vaccine development for both HPV-negative and EBV-positive tumors is limited in the absence of viable tumor-associated antigen targets. Furthermore, the heterogeneity and the relatively inactive immune landscape of these tumors stifles the efficacy of immunotherapy as a whole. ICI and therapeutic vaccines in this setting may serve as adjuncts to chemotherapy to be efficacious.

Future research efforts may also focus on the development of personalized tumor neoantigen vaccines, which offer a targeted response that is specific to the molecular signature of an individual tumor. This strategy may circumvent the lack of immunogenic and targetable tumor-associated antigens in these tumor types. Further investigation can elucidate whether current treatment paradigms can further enhance their efficacy—both in early- vs. late-stage disease.

### 6.4. Early but Promising Results in HPV-Positive Disease

There is a growing body of evidence to suggest potential benefit of therapeutic vaccines in the treatment of HPV-associated disease in combination with ICI, though still in its nascent stages. These tumors have demonstrated modest responses across multiple, novel platforms and greater clinical activity in combination with immunotherapy. However, their benefits remain limited despite their relatively greater density of TILs and more immunologically active landscape. This may indicate a possible level of immune function dysregulation at baseline in this patient population.

While immunocompetent individuals clear most acute HPV infections, a subset of patients progress to chronic infection and oncogenesis [106,107]. HPV infection is associated with local immunosuppression such as MHC downregulation and impaired cytokine signaling [108,109,110]. These same pathways may also limit the efficacy of vaccine therapy in this setting. Dedicated efforts to understand underlying immune dysregulation may benefit this patient population.

### 6.5. The Challenge of Tumor Heterogeneity

The overall mixed response of therapeutic vaccines underscores the complex anatomic and molecular heterogeneity of HNSCC and NPC. Tumors are driven by a variety of factors including viral infection, toxin exposure from alcohol or tobacco, and distinct genetic alterations resulting in genomic and phenotypic variability. Integration of therapeutic vaccines and choice of appropriate agent require thorough consideration of these contexts.

For example, while virally driven HNSCC is relatively more responsive to immunotherapy overall, the immune microenvironments between tumors differ significantly. Each microenvironmental subtype exhibits variable TIL enrichment, T-cell receptor diversity, plasma cell maturation patterns, and antibody isotype expression [111]. These differences likely contribute to variable to responses to immunotherapy and must be taken into account when designing clinical trials by site and interpreting the results [112].

Furthermore, treatment decisions are driven by the clinical context of early and resectable tumors versus RM disease. Most clinical trials for therapeutic vaccines are early-phase studies that have primarily focused on treating RM HNSCC and NPC. Most data available are preliminary, and only a limited number of trials have investigated vaccines for curative intent.

Overall, tumor heterogeneity and specific molecular profiling will continue to inform choice of treatment. Viral-associated tumors with highly conserved viral antigens, such as E6 and E7 for HNSCC and LMP1/2 for NPC, will likely benefit from peptide or nucleotide-based vaccine therapies. Personalized neoantigens may be suited for tumors that have high mutational burden but lack actionable vaccine targets, such as tobacco-associated HNSCC.

Immunotherapy and targeted cancer treatment is rapidly evolving. For each vaccine platform, future research in this space may focus on the appropriate clinical context, sequencing with respect to current paradigms, choice of ICI, potential combination with chemotherapy with or without radiation, ideal patient selection, and overall strategies to maximize durability and duration of response.

## 7. Conclusions

Therapeutic vaccines represent a promising but still developing strategy in the treatment of HNSCC and NPC. Currently, the strongest rationale and data for vaccine therapy exists for RM HPV-associated HNSCC in combination with ICIs. Several therapeutic candidates are now under evaluation for curative treatment of early-stage HNSCC. Effective vaccine delivery in HPV-negative HNSCC and EBV-positive NPC is still in its early stages.

The integration of therapeutic vaccines into current treatment paradigms is challenged by several factors including immune escape, a paucity of immunogenic targets, and tumor heterogeneity. Future advancement calls for robust phase 3 investigation, continued innovation, biomarker-driven development, and rational combinations that effectively complement existing treatment strategies.

## Data Availability

No new data were created or analyzed in this study. Data sharing is not applicable to this article.

## Supplementary Materials

The following supporting information can be downloaded at: https://www.mdpi.com/article/10.3390/vaccines14040321/s1, Table S1: Keyword Search.

## Appendix A. Therapeutic Vaccines for HPV-Positive HNSCC

**Vaccine (Trial, NCT)**	**Phase**	**N**	**Platform**	**Regimen Studied**	**Primary Endpoint(s)**	**Secondary Endpoints**	**Estimated Completion**
**Recurrent/Metastatic Disease—First-Line Treatment**							
ISA101 (NCT02426892) [34,35]	II	33	Peptide-based	ISA101plus nivolumab IV	**ORR:** CR 8.3%; PR 25%	**PFS at 3 yr:** 12.5 mo (4.3–36) **mOS:** 15.3 mo (10.6–27.2) **OS at 3 yr:** 12.5 mo (4.3–36)	Nov. 2021
ISA101b (OpcemISA, NCT03669718) [36]	II	194	Peptide-based	ISA101plus cemiplimab vs. placebo plus cemiplimab q3 wk	**ORR:** 25.3% vs. 22.9% (*p* = 0.735)	**mOS:** 15.8 vs. 26.9 mo; **mPFS:** 5.5 vs. 20.3 mo	Jun. 2025
ISA101b (NCT04398524) [37]	II	65	Peptide-based	ISA101b (4 doses) plus cemiplimab q3 wk	**ORR:** PR 11.5%; SD 50%; PD 30.8%	Not specified	Dec. 2024
PDS0101 (VERSATILE-002, NCT04260126) [38]	II	95	Peptide-based	Pembrolizumab IV plus PDS0101 SC	**ORR:** CPS ≥ 1: 35.8%; CPS 1–19: 28.1%; CPS ≥ 20: 47.6%	**PFS:** CPS ≥ 1: 30 mo (23.9–NE); CPS ≥ 1–19: 29.5 mo (15.3–NE); CPS ≥ 20: 39.3 mo (18.4–NE); **mOS:** CPS ≥ 1: 30 mo (23.9–NE); CPS ≥ 1–19: 29.5 mo (15.3–NE); CPS ≥ 20: 39.3 mo (18.4–NE)	May 2025
PDS0101 (VERSATILE-003, NCT06790966) [39]	III	351	Peptide-based	Pembrolizumab ± PDS0101	**OS:** In progress	**ORR, DCR, DOR, PFS:** In progress	Feb. 2029
PDS0101—positive NHS-IL12—positive M7824 (NCT04287868) [42]	I/II	51	Peptide-based	Arm 1 (HPV-associated malignancies): PDS0101plus NHS-IL-12 plus M7824 Arm 2 (Cervical Cancer with prior pelvic XRT): PDS0101 plus NHS-IL 12 plus M7824	**BOR:** Overall ORR 22% (CR 8.0%, PR 14.0%) HPV16-positive ORR 29.7%(CR 10.8%, PR 18.9%) HPV16-positive ICI-naïve ORR 62.5% (CR 25%, PR 37.5%)	**mDOR:** Arm 1: 10.6 mo (3.7–NA); Arm 2: 10.2 mo (NA–NA); **PFS:** Not specified; **OS:** Not specified	Jul. 2026
HB-201/HB-202 (NCT04180215) [48]	I/II	198	Viral vector	HB-201 or alternating HB-201/HB-202 ± pembrolizumab	**ORR:** CPS ≥ 1: 43% (3 CR, 9 PR); CPS ≥ 20: 59% (3 CR, 6 PR)	**DCR:** CPS ≥ 1: 71%; CPS ≥ 20: 88%	Jan. 2025
HB-201/HB-202 (NCT06373380) [49]	I	10	Viral vector	Alternating HB-201/HB-202	**OS:** In progress	**Adverse events:** In progress	Apr. 2027
PRGN-2009 (NCT04432597) [51]	I/II	39	Viral vector	PRGN-2009 ± bintrafusp alfa (M7824)	**Safety/RP2D:** ORR 0% (monotherapy); ORR 20% (combination)	**mOS:** 24 mo (9.6–NR); **Phase II endpoints:** OS, PFS, DOR, ORR ongoing	Jan. 2026
MSB0011359C (NCT02517398) [53]	I	600	Viral vector	Bintrafusp alfa IV q2 wk	**BOR (IRC):** ORR 13% (4–29%)	**PR:** 4 pts; **SD:** 4 pts; **DCR:** 34% (12–43%); **Investigator ORR:** 16% (PR in 5 pts)	May 2022
BNT113 (AHEAD-MERIT, NCT04534205) [56]	II/III	285	Nucleic acid	Pembrolizumab ± BNT113	**mPFS:** 3.9 mo (2.1–12.9)	**mOS:** 22.6 mo (11.8–NE); **PFS at 6 mo:** 42%; **PFS at 12 mo:** 28%; **ORR, DOR, DCR:** In progress	Apr. 2029
INO-3112 (NCT03162224) [59]	I/II	35	Nucleic acid	Arm 1 1L RM platinum non-refractory: INO-3112 EP—positive durvalumab Arm 2 1L platinum-refractory: Same as Arm 1 Arm 3 2L-positive RM: Same as Arm 1	**ORR:** Up to 41.7% (irRECIST)	**DCR:** Up to 53.3%; **mPFS:** Up to 9.5 mo; **OS:** Up to 29.2 mo; **Anti-drug antibodies:** 1 pt (9.1%)	Mar. 2021
**Recurrent/Metastatic Disease—≥2L Treatment**							
CUE-101 (NCT03978689) [70]	I	85	T-cell engager	CUE-101 ± pembrolizumab	**DLT/PK:** PR in 6 pts	**mOS:** 24.4 mo (9.1–NA)	Jan. 2026
**Neoadjuvant/Induction Therapy**							
PDS0101 (NCT05232851) [38]	I/II	20	Peptide-based	PDS0101 ± pembrolizumab (2 cycles)	**ctHPV-DNA ≥50% decline:** 0% vs. 50%	**PFS, OS, RR:** In progress	Oct. 2026
HB-201/HB-202 (TARGET-HPV, NCT05108870) [54]	I/II	98	Viral vector	HB-201/HB-202—positive carboplatin/paclitaxel	**DRR:** 81%	**Pathologic response:** NED in all surgical pts; **PFS/OS:** In progress; **Plasma HPV-DNA correlation:** In progress	Jan. 2026
**Definitive Therapy**							
ISA101b (NCT04369937) [55]	II	18	Peptide-based	IMRT—positive cisplatin—positive pembrolizumab—positive ISA101b	**2 yr PRS:** In progress	**PFS (≤3 yr), OS:** In progress	Jan. 2026
Unless otherwise noted, primary and secondary outcomes are reported as experimental arm vs. placebo arm and 95% CI noted in parentheses.							
