# Supplementary material for: Therapeutic Vaccines for Head and Neck Squamous Cell Carcinoma and Nasopharyngeal Carcinoma
Source: Vaccines (Basel). 2026 Apr 3;14(4):321. doi: 10.3390/vaccines14040321 (PMC13119602; doi:10.3390/vaccines14040321)
Supplement: Supplementary file 1 [file vaccines-14-00321-s001.zip › vaccines-4182600-supplementary.pdf]

Topic	Therapeutic Vaccines in HNSCC and NPC
Keywords	head and neck squamous cell carcinoma (HNSCC); nasopharygeal carcinoma; Epstein–Barr virus (EBV); human papilloma virus (HPV); immunotherapy; therapeutic vaccine; mRNA; peptide; viral vector; T-cell engager; nucleic acid; personalized vaccine; tumor-associated antigen; neoantigen
Timeline	4 weeks
Databases Searched	PubMed (NCBI), ClinicalTrials.gov
Search Created by	Michael Baliton,MD, UC Irvine Library
